# Polycrystalline Diamond as a Potential Material for the Hard-on-Hard Bearing of Total Hip Prosthesis: Von Mises Stress Analysis

**DOI:** 10.3390/biomedicines11030951

**Published:** 2023-03-20

**Authors:** Muhammad Imam Ammarullah, Rachmad Hartono, Toto Supriyono, Gatot Santoso, S. Sugiharto, Muki Satya Permana

**Affiliations:** 1 Department of Mechanical Engineering, Faculty of Engineering, Universitas Pasundan, Bandung 40153, West Java, Indonesia; 2 Biomechanics and Biomedics Engineering Research Centre, Universitas Pasundan, Bandung 40153, West Java, Indonesia

**Keywords:** hard-on-hard, total hip prosthesis, von Mises stress, finite element method, polycrystalline diamond

## Abstract

Due to polymeric wear debris causing osteolysis from polymer, metal ions causing metallosis from metal, and brittle characteristic causing fracture failure from ceramic in the application on bearing of total hip prosthesis requires the availability of new material options as a solution to these problems. Polycrystalline diamond (PCD) has the potential to become the selected material for hard-on-hard bearing in view of its advantages in terms of mechanical properties and biocompatibility. The present study contributes to confirming the potential of PCD to replace metals and ceramics for hard-on-hard bearing through von Mises stress investigations. A computational simulation using a 2D axisymmetric finite element model of hard-on-hard bearing under gait loading has been performed. The percentage of maximum von Mises stress to respective yield strength from PCD-on-PCD is the lowest at 2.47%, with CoCrMo (cobalt chromium molybdenum)-on-CoCrMo at 10.79%, and Al_2_O_3_ (aluminium oxide)-on-Al_2_O_3_ at 13.49%. This confirms that the use of PCD as a hard-on-hard bearing material is the safest option compared to the investigated metal and ceramic hard-on-hard bearings from the mechanical perspective.

## 1. Introduction

Over the last few decades, polymer, metal, and ceramic have been the available material options for application consideration in the bearing of total hip prosthesis [1,2,3]. Each material has its advantages, polymer is capable of effective damping due to impact loads from unexpected activities [4]; metals are suitable for active younger users with high-level intensity [4]; ceramic has a smooth surface with a low coefficient of friction [5]. However, there are several serious problems found in the use of these three materials from a clinical standpoint. For example, polymer produces polymeric wear debris, which trigger osteolysis [6]; metal can release metal ions that enter the body tissues, causing metallosis [7]; ceramic has a disadvantage of fracture failure [8].

The problems of the three previous available material options have prompted the availability of new materials for the bearing of total hip prosthesis as the solution to the conditions faced in material selection. From this condition, polycrystalline diamond (PCD) has been proposed as the new option for bearing material. PCD is a material consisting of several diamond crystals arranged together [9]. Several characteristics such as its excellent hardness [10], extreme wear resistance [11], low coefficient of friction [12], superior toughness [13], and good biocompatibility [14] make it a potential material for bearing applications in a total hip prostheses. Another advantage of PCD is its good dimensional stability and resistance to deformation and mechanical damage [15].

**Figure 1:**
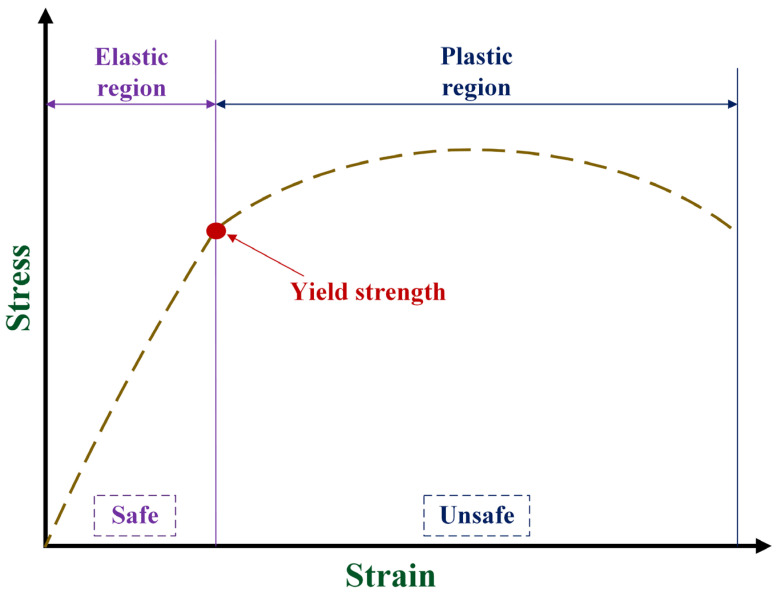
Stress-strain curve [23]. Bearing of total hip prosthesis is indicated to be safe when the von Mises stress does not exceed the yield strength, which indicates that it is still in the elastic region of stress-strain curve.

It is necessary to ensure the safety of the bearing in a total hip prosthesis when used to accommodate the various activities conducted by the implant users to reach long-term service life. Ensuring medical implant safety can be achieved clinically or experimentally, but both of these require a relatively long time and are not cheap, making computational simulation-based studies a rational option [16,17,18]. Von Mises stress is an indicator for measuring material failure [19] that has been observed in computational simulation studies on the bearing of total hip prosthesis. When von Mises stress occurs in the bearing to accommodate the implant user’s activities, the user has exceeded the yield strength of the bearing material; this causes failure due to the loss of its elastic properties, and it cannot return to its original form [20], as shown in Figure 1. Changes in the bearing caused by the loss of its elastic properties can affect several problems in total hip prosthesis ranging between triggering dislocations, poor lubrication conditions, and non-smooth articulations. If these conditions have occurred, the replacement with new bearings through revision surgery is unavoidable which is detrimental to the implant user, both from health [21] and economic [22] perspectives.

A computational study of the von Mises stress on the bearings of total hip prosthesis via finite element method has been previously reported by Saputra et al. [24] by who studied different combinations of bearing materials, both hard-on-hard and hard-on-soft such as Al_2_O_3_ (aluminium oxide)-on-Al_2_O_3_, Al_2_O_3_-on-SS 316L (stainless steel 316L), CoCr (cobalt chromium)-on-CoCr, CoCr-on-SS 316L, and SS 316L-on-UHMWPE (ultra-high molecular weight polyethylene). Furthermore, Kalayarasan et al. [25] also analyzed the von Mises stress on CoCr-on-UHWMPE, CoCr-on-CoCr, CoCr-on-CoCrMo (cobalt chromium molybdenum), and CoCr-on-Al_2_O_3_ bearings. With more focus on hard-on-soft bearings, a similar investigation was carried out by Shankar et al. [26] through a SS 316L-on-UHMWPE, CoCr-on-UHMWPE, CoCrMo-on-UHMWPE, and Titanium-on-UHMWPE bearings investigation. Previous research conducted on von Mises stress on the bearing of total hip prosthesis has not focused on exploring hard-on-hard bearings, nor has PCD-on-PCD been involved in von Mises stress analysis. This research gap needs to be filled by investigating the von Mises stress of PCD-on-PCD bearing in comparison with other hard-on-hard bearings, such as metal-on-metal and ceramic-on-ceramic to ascertain the potential application of PCD in hard-on-hard bearing.

The present computational study aims to assess the safety of PCD as potential material for hard-on-hard bearings to replace metal and ceramic materials. This is achieved by evaluating the von Mises stress in metal-on-metal, ceramic-on-ceramic, and PCD-on-PCD bearings, and then comparing it to the respective yield strength of the bearing materials. The finite element model of hard-on-hard bearings is established for understanding the bearing safety based on the mechanical perspective.

## 2. Materials and Methods

### 2.1. The Parameter in Finite Element Simulation of Hard-on-Hard Bearings

In the present study, the geometry of a hard-on-hard bearing of total hip prosthesis, both for femoral head and acetabular cup component, use common geometry adopted in the bearings of total hip prosthesis [27], presented in Table 1. The hard materials investigated in the present study, include CoCrMo, Al_2_O_3_, and PCD, which are used in both acetabular cup and femoral head components, forming metal-on-metal (CoCrMo-on-CoCrMo), ceramic-on-ceramic (Al_2_O_3_-on-Al_2_O_3_), and diamond-on-diamond (PCD-on-PCD) bearings. The Young’s modulus, Poisson’s ratio, and coefficient of friction from the hard-on-hard bearings in the present computational simulation are shown in Table 2. assuming that they become homogeneous, isotropic, and linear elastic [28]. The Young’s modulus and Poisson’s ration is an input parameter used to describe the mechanical properties of hard materials during the computational simulation process [29]. In addition, the coefficient of friction is an input parameter used to describe the asperity between femoral head and acetabular cup contact surfaces [30].

### 2.2. Finite Element Model of Hard-on-Hard Bearings

**Figure 2:**
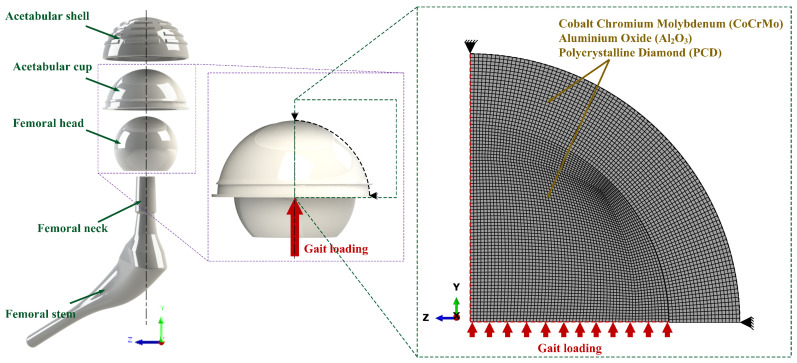
Component consideration and finite element model of hard-on-hard bearings.

The computational simulation of the von Mises stress of the hard-on-hard bearings was conducted using ABAQUS CAE version 6.14-1 (Dassault Systèmes, Vélizy-Villacoublay, France) with the consideration of two main components, namely acetabular cup and femoral head to simplify the modelling as shown in Figure 2. The finite element model of the hard-on-hard bearings adopts a 2D axisymmetric, as previous researched by Saputra et al. [24]. The definition of contact is configured of the contact surface of femoral head as master surface, while the contact surface of acetabular cup as slave surface. The finite element model was discretized with 5500 four-node axisymmetric elements (CA4X), with 2000 CAX4 elements for the acetabular cup component, and the remaining elements being used for the femoral head component. These were obtained through element convergence studies before the von Mises stress analysis was conducted (described in Section 3.1. Convergence Study). For the boundary conditions, gait loading was applied to the lower section of femoral head, with only femoral head being allowed to move vertically concentric to acetabular cup, and acetabular cup was immobilized [33]. In addition, the presence of synovial fluid lubrication during contact is ignored; only dry contact occurs. However, the effect of lubrication and surface roughness is incorporated into the coefficient of friction parameter [34].

**Figure 3:**
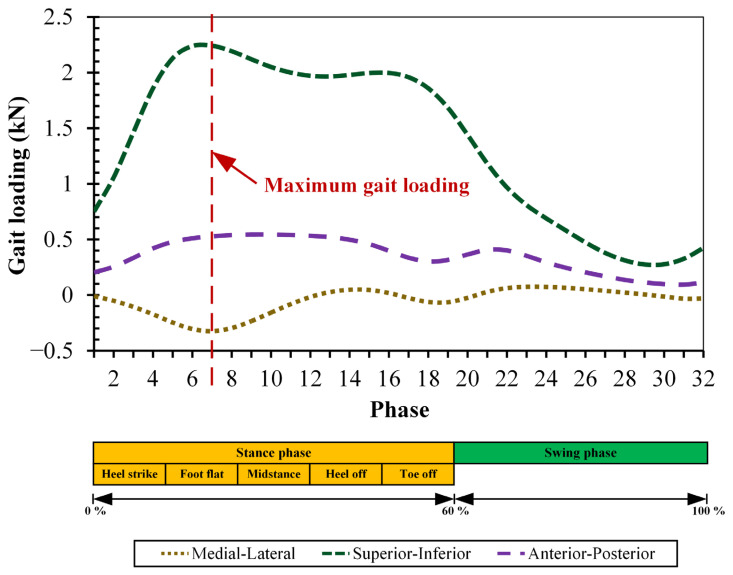
Gait loading simulated on the hard-on-hard bearing of total hip prosthesis [35].

### 2.3. Gait Loading

With the rationalization that users of a total hip prosthesis perform most common of their activities by walking normally, the loading conditions in the present computational simulation of hard-on-hard bearings of total hip prosthesis simulate normal walking activity [36]. The gait loading in the current study, as shown in Figure 3 only considers the vertical force without the range of motion as was conducted by Jamari et al. [35]. This is because the finite element model of hard-on-hard bearings currently uses 2D axisymmetric, making it impossible to adopt triaxial motion during gait loading [37]. For the convenience of the computational simulation procedures, the gait loading is divided into 32 phases as referred from previous study [27,34,38] consisting of two main groups, namely the stance phase (60% of gait loading) and the swing phase (40% of gait loading) [39]. Peak loading occurs in the 7th phase (stance phase) of 2326.09 N [35].

## 3. Results and Discussion

### 3.1. Convergence Study

**Figure 4:**
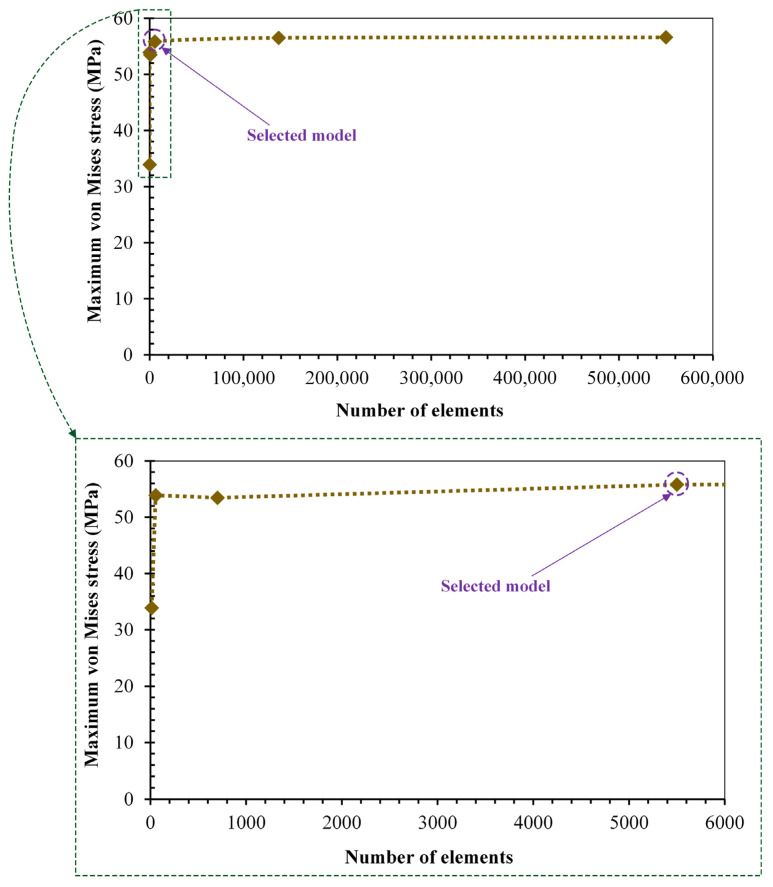
Convergence study of CoCrMo-on-CoCrMo bearing from von Mises stress result.

The number of elements used for the von Mises stress investigation of the hard-on-hard bearings of total hip prosthesis was selected based on the convergence studies of six identical models with different elements, including coarse elements (small number of elements) and fine elements (large number of elements) presented in Figure 4. It aims to select hard-on-hard bearing models with an optimal number of elements, which does not use too many elements in order to not burden the computational load, whilst still being able to provide accurate von Mises stress results [40]. The convergence study was conducted by analyzing the highest von Mises stress (at the 7th phase of gait loading) on the CoCrMo-on-CoCrMo bearings. The 4th model has been chosen from the convergence study with the number of 5500 CAX4 elements (2000 CAX4 elements for acetabular cup and 3500 CAX4 elements for femoral head), as is explained in Figure 3. This is because the von Mises stress obtained from the 4th model is 55.8 MPa with a difference of 0.79 MPa (under 2%) compared to 6th model, and had the highest number of elements.

### 3.2. Validation of von Mises Stress Results

**Figure 5:**
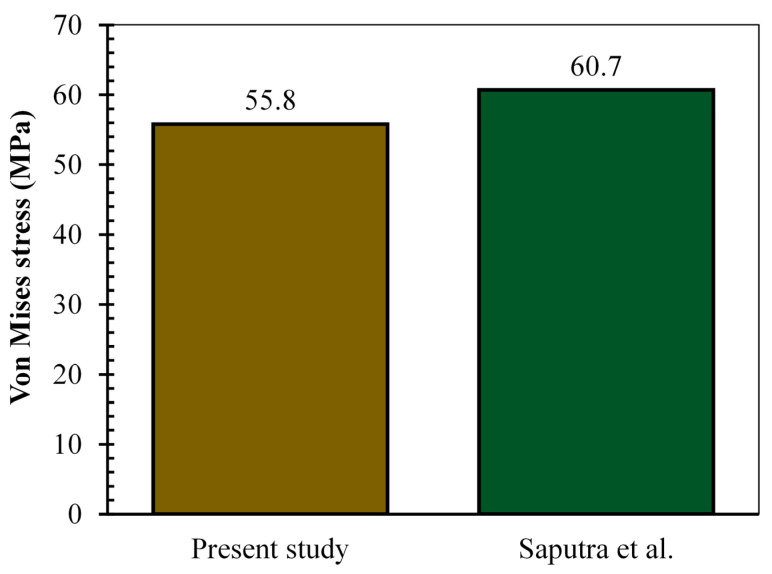
Present von Mises stress result validation of CoCrMo-on-CoCrMo bearing of total hip prosthesis with similar previous von Mises stress investigation performed by Saputra et al. [24].

The current von Mises stress results from the computational simulation of hard-on-hard bearings of total hip prosthesis need to be validated to ensure the accuracy of the results obtained in previous studies under identical conditions and parameters [41]. The highest von Mises stress (in the 7th phase of the gait loading) for the CoCrMo-on-CoCrMo bearing was validated by the previous research by Saputra et al. [24] presented in Figure 5. The difference of the highest von Mises stress result from both of them is 4.9 MPa indicating a difference of around 8.78%. The von Mises stress results in the current study have been validated due to differences in the acceptable range, which is under 10% [35].

### 3.3. Von Mises Stress Investigation

**Figure 6:**
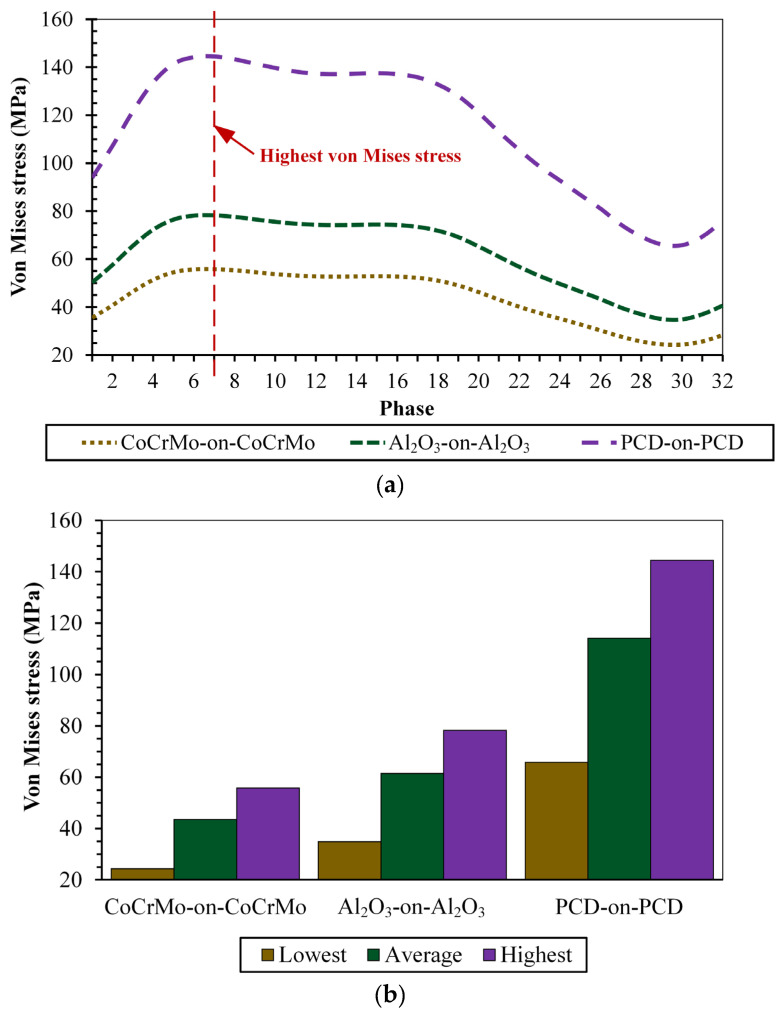
Von Mises stress of hard-on-hard bearings: (**a**) maximum value in full 32 phases of gait loading and (**b**) lowest, average, and highest value.

Figure 6a presents a comparison of the maximum von Mises stresses during each phase of gait loading divided into 32 phases for three types of hard-on-hard bearings consisting of CoCrMo-on-CoCrMo, Al_2_O_3_-on-Al_2_O_3_, and PCD-on-PCD. The value of the von Mises stress changes in each phase due to the difference in the resultant force applied, where the highest von Mises stress is in the 7th phase as this phase has a peak gait loading, and vice versa for the lowest von Mises stress found in the 30th phase because of the lowest gait loading. The comparison of the lowest, average, and highest von Mises stress for the studied hard-on-hard bearings is presented in Figure 6b.

**Figure 7:**
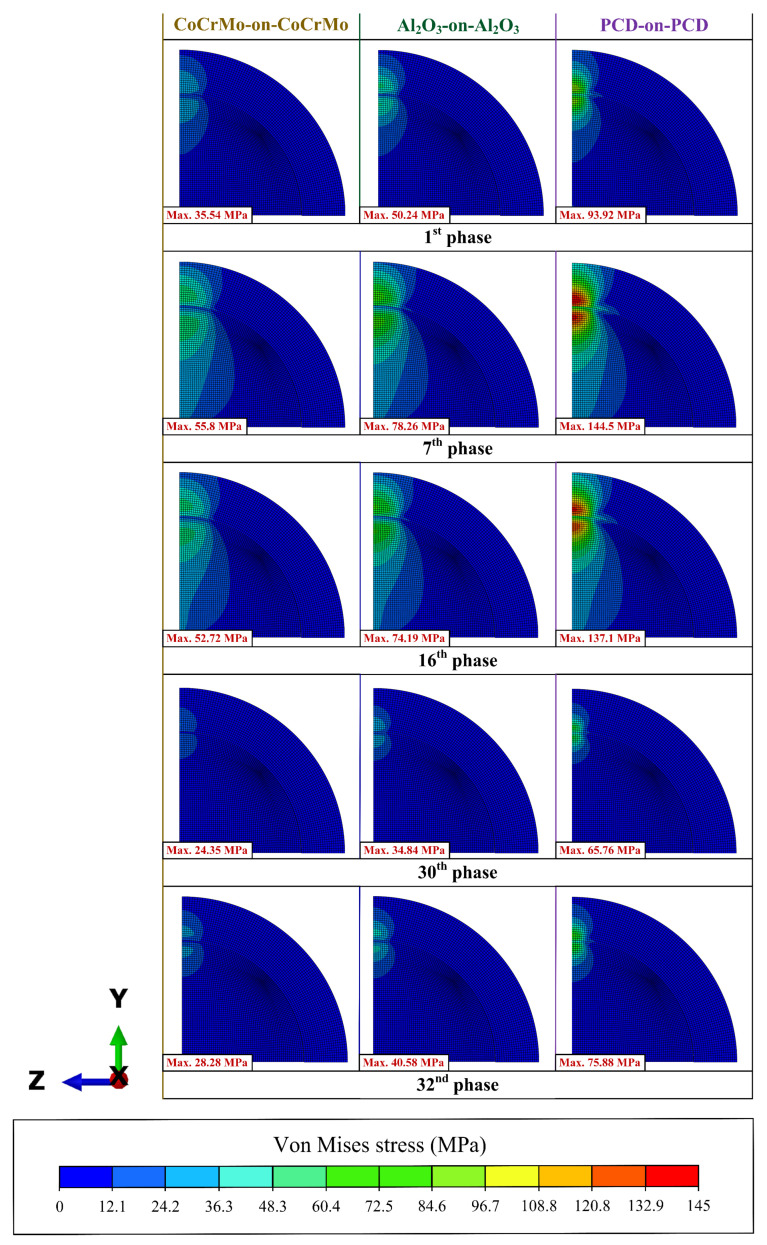
Contour of von Mises stress of hard-on-hard bearings during representative phases.

The distribution contour of the von Mises stress from hard-on-hard bearings is shown in Figure 7. Five representative phases were selected out of the 32 gait loading phases to present the von Mises stress contour of the hard-on-hard bearings in the current study referring to the previous report by Ammarullah et al. [42] consisting of 1st (initial of gait loading), 7th (peak of gait loading), 16th (mid of gait loading), 30th (lowest of gait loading), and 32nd (end of gait loading) phases. It can be seen that the intensity and distribution of the von Mises stress contour will increase and widen as the von Mises stress value increases on the hard-on-hard bearings. As the overall von Mises stress of PCD-on-PCD is the highest compared to CoCrMo-on-CoCrMo and Al_2_O_3_-on-Al_2_O_3_, the intensity and contour distribution are the highest and widest. The von Mises stress distribution contour in the selected phases shows a similar pattern to the contours found in the center of contact area between acetabular cup and femoral head. This is because the current computational simulation model does not consider the range of motion and only forces that move vertically, so the movement of the femoral head will be concentric on the acetabular cup.

**Figure 8:**
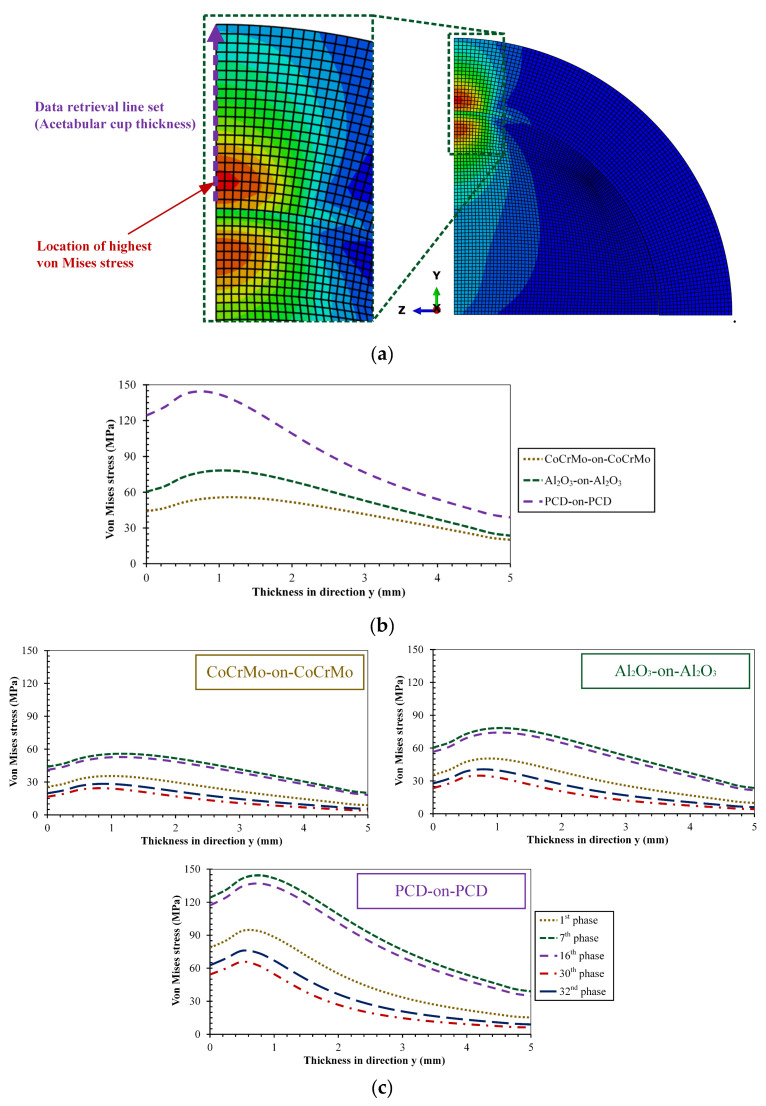
Von Mises stress profile as a function of acetabular cup thickness on hard-on-hard bearings: (**a**) Data retrieval line set, (**b**) profile at peak loading, and (**c**) profile at selected phases.

The von Mises stress on the hard-on-hard bearings is studied further by looking at the profile in acetabular cup thickness. This was conducted by observing the von Mises stress on a data retrieval line set on the acetabular cup thickness in the direction y as shown in Figure 8a. Meanwhile, the von Mises stress profile of the acetabular cup thickness at the peak loading and selected phases is described in Figure 8b and Figure 8c respectively. The study of the relationship between von Mises stress and acetabular cup thickness is vital to do because the highest von Mises stress is not located at the bearing interface, but in the bulk area, where it has a risk of losing its elastic properties first [38]. Recognizing the areas, in which the material’s elastic properties are most likely to be lost first contributes to avoiding future surface damage to the bearing of total hip prosthesis.

The maximum value of the von Mises stress (in the 7th phase of gait loading) for the three hard-on-hard bearings is shown in Table 3, where from highest to lowest it belongs to CoCrMo-on-CoCrMo, Al_2_O_3_-on-Al_2_O_3_, and PCD -on-PCD. The von Mises stress increased by 55.8 MPa (158.06%) when CoCrMo-on-CoCrMo was compared to PCD-on-PCD and increased by 66.24 MPa (84.64%) when Al_2_O_3_-on-Al_2_O_3_ was compared to PCD-on-PCD. The significant increase in the von Mises stress value of PCD-on-PCD compared to the other hard-on-hard bearings is due to the high hardness of the PCD material [43], indicated by the mechanical properties from Young’s modulus of 900 GPa [32], which is high in comparison to that of CoCrMo of 210 GPa [27] and Al_2_O_3_ of 375 GPa [31].

The present computational simulation results confirm that the von Mises stress of all the hard-on-hard bearings are still below the yield strength as presented in Table 3. This shows that explains in real conditions, the hard-on-hard bearings will not failure due to undergoing plastic deformation during walking activities conducted by the implant users. Therefore, even under the peak of gait loading (7th phase), the material of the hard-on-hard bearings retains its elastic properties and can return to its original shape after being subjected to a gait loading. However, the safest hard-on-hard bearings, from the mechanical perspective is PCD-on-PCD because the maximum von Mises stress value is only 2.47% of the PCD’s yield strength. Although the von Mises stress value of PCD-on-PCD is the highest compared to the metals and ceramic hard-on-hard bearings used in the current study, the Yield strength of PCD is very high at 5849.7 MPa [44] making the percentage of the maximum von Mises stress to the yield strength very small. In terms of the safest bearings after PCD-on-PCD, CoCrMo-on-CoCrMo and Al_2_O_3_-on-Al_2_O_3_ had the percentage of maximum von Mises stress to respective yield strength, at 10.79% for CoCrMo-on-CoCrMo and 13.49% for Al_2_O_3_-on-Al_2_O_3_. The ceramic-on-ceramic bearings were the most unsafe hard-on-hard bearings from a mechanical perspective. The explanation is rational because of the brittle nature of the ceramic, which puts this bearing at risk of failure due to fracture when subjected to high loads [45].

### 3.4. Challenges and Opportunities on PCD-on-PCD bearing

There are three common materials available that were previously widely used in the bearings of total hip prostheses; metals [46], ceramics [47], and polymers [48]. These materials would generally be fabricated with conventional machining [49]. However, it is not possible to fabricate PCD with conventional machining that requires electrical discharge machining (EDM) [50] or laser machining [51] due to the high level of fabrication difficulty as it has superior hardness compared to metals, ceramics, and polymers [52]. Fabrication methods using EDM and laser machining are very expensive and require sophisticated fabrication equipment when compared to conventional machining; this renders PCD far less affordable compared to metals, ceramics, and polymers [53]. This is exacerbated by the availability of the material; PCD requires diamond crystal as a raw material, which is difficult to obtain, as well as bearing expensive and not widely available in the commercial market. This situation is very different when compared to metals, ceramics, and polymers whose raw materials are easy to obtain, relatively cheaper, and widely available in the commercial market. The widespread use of PCD-on-PCD bearings in total hip prostheses quite difficult at present, especially in developing countries due to low purchasing power for expensive medical devices and the availability of qualified sophisticated fabrication equipment. However, with the development of technology, PCD-on-PCD bearings will potentially become widely available in the future, especially considering all the advantages of PCD materials compared to metals, ceramics, and polymers, with PCD-on-PCD providing a higher performance compared to the other available bearings, both hard-on-hard (metal-on-metal and ceramic-on-ceramic) [54] and hard-on-soft (metal-on-polymer and ceramic-on-polymer) [55]. The explanation is also supported by the current results of the von Mises stress investigation, which shows that PCD-on-PCD is the safest bearing from a mechanical standpoint compared to CoCrMo-on-CoCrMo and Al_2_O_3_-on-Al_2_O_3_.

### 3.5. Limitations of the Current Computational Research

Regarding the limitation of the current computational research, some crucial remarks must be made. From the perspective of the adopted gait loading, the loading only adopts acting forces, not the range of motion, which limits the movement only in the vertical direction. The simplification does not perfectly reflect the realistic situation in which the implant user performs walking activity [56]. From the coefficient of friction, the used constant value is chosen. However, the coefficient of friction changes over time when contact occurs between femoral head and acetabular cup, representing changes in the lubrication condition, surface roughness, and wear [57]. Additionally, the present finite element modelling only employs a 2D form to investigate von Mises stress on hard-on-hard bearings, which are less accurate than 3D models. It was conducted to reduce the amount of calculation time needed [58]. Next, only femoral head and acetabular cup components are taken into account. In contrast, considering other components, such as pelvic bone and acetabular shell, results in a more realistic modelling scenario [59]. In terms of the materials assumption, the present finite element model assumes a purely elastic material, without considering plastic behavior on CoCrMo, Al_2_O_3_, and PCD. An elasto-plastic model needs to be considered in a bearing investigation of total hip prosthesis in order to present a more realistic modelling as established by Teoh et al. [60]. Lastly, the current von Mises stress investigation does not validate the elevant experimental testing, but only validate the with previous similar von Mises stress results performed by Saputra et al. [24]. Future study must solve these drawbacks as the present limitations can undoubtedly influence the computational simulation outcomes.

## 4. Conclusions

Finite element simulation has been performed on CoCrMo-on-CoCrMo, Al_2_O_3_-on-Al_2_O_3_, and PCD-on-PCD bearings of total hip prosthesis to investigate the von Mises stress using a 2D axisymmetric model. The von Mises stress outcomes in the current study show the highest value in the 7th phase of gait loading for metal, ceramic, and diamond hard-on-hard bearings. PCD-on-PCD shows the highest maximum von Mises stress compared to the other investigated hard-on-hard bearings. However, when the maximum von Mises stress is compared with the respective yield strength, PCD-on-PCD is the lowest. The result confirms that PCD as a potential material for hard-on-hard bearings is the safest material from a mechanical point of view compared to CoCrMo and Al_2_O_3_, thus implying PCD-on-PCD as a promising hard-on-hard bearing in the future. It is also supported by the superior biocompatibility of PCD making the application of the material safe for the human body from a biomedical perspective.

## Figures and Tables

**Table 1:** Geometry of hard-on-hard bearings.

Parameter	Size (mm)
Radius of femoral head	14
Radial clearance	0.05
Thickness of acetabular cup	5

**Table 2:** Young’s modulus, Poisson’s ratio, and coefficient of friction from hard-on-hard bearings.

Hard-on-Hard Bearing	Young’s Modulus (GPA)	Poisson’s ratio (-)	Coefficient of Friction (-)	Reference
CoCrMo-on-CoCrMo	210	0.3	0.2	[27]
Al_2_O_3_-on-Al_2_O_3_	375	0.3	0.1	[31]
PCD-on-PCD	900	0.1	0.1	[32]

**Table 3:** Percentage of maximum von Mises stress to respective yield strength from hard-on-hard bearings.

Hard-on-Hard Bearing	Maximum von Mises Stress (MPa)	Yield Strength (MPa)	Percentage of Maximum von Mises Stress to Respective Yield Strength (%)
CoCrMo-on-CoCrMo	55.8	517 [25]	10.79
Al_2_O_3_-on-Al_2_O_3_	78.26	580 [26]	13.49
PCD-on-PCD	144.5	5849.7 [44]	2.47

## Data Availability

The data presented in this study are available on request from the corresponding author.
